# A phase II trial of intermittent nivolumab in patients with metastatic renal cell carcinoma (mRCC) who have received prior anti-angiogenic therapy

**DOI:** 10.1186/s40425-019-0615-z

**Published:** 2019-05-16

**Authors:** Moshe C. Ornstein, Laura S. Wood, Brian P. Hobbs, Kimberly D. Allman, Allison Martin, Michael Bevan, Timothy D. Gilligan, Jorge A. Garcia, Brian I. Rini

**Affiliations:** 1 Department of Hematology and Medical Oncology, Cleveland Clinic Taussig Cancer Institute, 9500 Euclid Avenue, CA-60, Cleveland, OH 44195 USA; 2 Quantitative Health Sciences and Taussig Cancer Institute, Cleveland Clinic, 9500 Euclid Avenue, Cleveland, OH 44195 USA

**Keywords:** Renal cell carcinoma, Kidney cancer, Immunotherapy, Nivolumab, Treatment-free interval, Checkpoint inhibitor

## Abstract

**Background:**

Nivolumab is approved for mRCC patients who have received prior anti-angiogenic therapy but the duration of therapy required for sustained clinical benefit is unknown. A phase II clinical trial to investigate the feasibility of intermittent nivolumab dosing was conducted.

**Methods:**

Patients ≥18 years of age with mRCC who were previously treated with at least one antiangiogenic therapy were eligible. Patients were treated with nivolumab for twelve weeks. Patients who had RECIST PD were removed from the trial. Patients who did not initially achieve ≥10% reduction in tumor burden (TB) continued nivolumab per standard of care. Patients with ≥10% TB reduction entered a treatment-free observation phase with re-imaging every 12 weeks. Nivolumab was restarted in patients with a ≥ 10% TB increase and again held with TB reduction ≥10%. This intermittent nivolumab dosing continued until RECIST PD while on nivolumab. The primary objective was feasibility of intermittent nivolumab, defined as the proportion of patients eligible for intermittent therapy who elect to receive intermittent nivolumab. Intermittent nivolumab would be considered “feasible” if the acceptance rate was ≥80%. Forty patients provides > 95% power with 0.05 type I error, assuming a null acceptance rate of 50%. With the approval of the combination of ipilimumab/nivolumab (April 2018) in front-line mRCC, this cohort was closed prior to completed pre-planned approval.

**Results:**

Of the 14 patients enrolled, 13 (93%) were male with a median age 65. All had a prior nephrectomy and 12 (86%) were intermediate-risk by IMDC criteria. Five patients (36%) met the criteria for the intermittent phase of the trial (median TB decrease 46%) and all agreed to intermittent therapy. With a median follow-up of 48 weeks, only one patient restarted therapy. The four remaining patients have a sustained response for a median of 34 weeks (range, 16–53) off therapy. No patients developed RECIST PD while off therapy.

**Conclusions:**

This prospective experience of intermittent nivolumab dosing in mRCC supports further investigation of intermittent immunotherapy dosing strategies in RCC.

**Trial registration:**

NCT03126331 (Intermittent Nivolumab in Metastatic Renal Cell Carcinoma Patients; Date of registration 4/27/2017; https://clinicaltrials.gov/ct2/show/NCT03126331).

## Introduction

Immunotherapy with checkpoint inhibitor (CPI) antibodies that target programmed cell death 1 (PD-1), programmed cell death ligand 1 (PD-L1), and cytotoxic T-lymphocyte antigen 4 (CTLA-4) are currently approved for the treatment of patients with metastatic renal cell carcinoma (mRCC). The anti-PD-1 agent nivolumab is approved for patients with previously-treated mRCC and the combination of nivolumab and ipilimumab (anti-CTLA-4) is approved for patients with treatment-naïve intermediate- and poor-risk mRCC [1, 2].

In addition to favorable toxicity profiles compared to prior standard of care mRCC agents such as vascular endothelial growth factor receptor (VEGFR) inhibitors, a key benefit of CPI therapy is the ability for patients to achieve long term durable responses. However, an unanswered question with the use of CPIs is the duration of therapy necessary to achieve and maintain durable responses. Analyses of the clinical trials which lead to the approval of nivolumab monotherapy in mRCC as well as the combination of ipilimumab/nivolumab demonstrate that a subset of patients can sustain durable responses to therapy following treatment discontinuation [1–5]. These treatment-free intervals (TFI) are critical as they limit cumulative physical and financial toxicity.

Prospective data investigating TFIs in mRCC lacking. A phase II trial investigating the feasibility of intermittent therapy in mRCC patients treated with nivolumab was therefore conducted (NCT03126331).

## Methods

### Study design and treatment

Patients ≥18 year old with mRCC of any histology who received at least one prior anti-angiogenic therapy were included. Patients were treated with nivolumab for twelve weeks per standard of care dosing (240 mg every 2 weeks or 480 mg every 4 weeks). Patients who had RECIST PD were removed from the trial. Patients who did not initially achieve ≥10% reduction in tumor burden (TB) continued nivolumab per standard of care. Patients with ≥10% reduction in TB entered a treatment-free observation phase with re-imaging every 12 weeks. Nivolumab was re-initiated in those patients with a ≥ 10% TB increase and again held with TB reduction ≥10%. This intermittent nivolumab dosing continued until RECIST PD while on nivolumab. Patients who did not achieve a 10% TB reduction at the time of the second scans (24 weeks after starting treatment), were removed from the protocol and continued nivolumab per standard of care.

### Objectives and statistical considerations

The primary objective was feasibility of intermittent nivolumab, which was defined as the proportion of patients eligible for intermittent therapy who elect to receive intermittent nivolumab. Intermittent nivolumab would be considered “feasible” if ≥80% of patients eligible for intermittent therapy, and “not feasible” is the acceptance rate was < 50%. Forty patients provides > 95% power with 0.05 type I error, assuming a null acceptance rate of 50%. Continuous variables are reported by range, sample median, and interquartile range (IQR). Statistical estimation of progression-free survival (PFS) used the Kaplan-Meier (KM) method. The study protocol and consent were approved by the Cleveland Clinic Institutional Review Board (IRB # 17–586).

### Study update

With the approval of the combination of ipilimumab/nivolumab (April 2018) in front-line mRCC and a host of other frontline immunotherapy combination trials, this cohort was closed prior to completed pre-planned approval due to limited use of nivolumab monotherapy [2]. A separate cohort is opening to investigate the role of intermittent therapy in patients who receive ipilimumab/nivolumab.

## Results

Fourteen patients with mRCC were included of which 13 (93%) were male with a median age of 65 (range, 57–72; IQR 10). All had a prior nephrectomy, 13 (93%) had clear-cell histology, 13 (93%) had KPS ≥ 80%, and 12 (86%) were intermediate-risk by IMDC criteria. Metastatic sites were typical for mRCC. Twelve patients (86%) received only one prior anti-angiogenic therapy (Table 1).

**Table 1:** Patient characteristics and prior therapy

Characteristic	N (%)
Median Age (range)	65 (57–72)
Gender	
Male	13 (93%)
Female	1 (7%)
Histology	
Clear cell	13 (93%)
Papillary	1 (7%)
ECOG	
0	9 (64%)
1	5 (36%)
IMDC risk group	
Favorable	1 (7%)
Intermediate	12 (86%)
Poor	1 (7%)
Metastatic sites	
Lymph nodes	8 (57%)
Bone	8 (57%)
Lung	7 (50%)
Liver	3 (21%)
Number or prior therapies	
1	12 (86%)
2	1 (7%)
3	1 (7%)
Most recent therapy	
Sunitinib	8 (57%)
Axitinib	3 (21%)
Pazopanib	2 (14%)
HIF-2a inhibitor	1 (7%)
Best response to most recent therapy^a^	
PR	3 (21%)
SD	9 (64%)
PD	1 (7%)
Median duration on most recent therapy (months)^a^	18 (3–100)

^a^Missing for one patient

In total, the objective response rate was 29%; 4 (29%) patients achieved a PR, 6 (43%) had SD, and 4 (29%) had primary RECIST PD. With a median follow-up of 6.01 months (range 0.92–15; IQR 7.57), 10 of the 14 patients experienced progression. The median PFS for all patients was 7.97 months (95% CI 5.42 – not estimable). Five patients (36%) met the pre-specified criteria to enter the intermittent phase of the trial and all agreed to intermittent therapy. The median TB decrease at the time of entering the intermittent phase was 46% (range, 22–91%; IQR 14). Four of the patients had the necessary TB decrease (≥ 10%) to qualify for intermittent therapy after 12 weeks of therapy and one patient achieved this TB threshold after receiving nivolumab for 24 weeks. Among 9 patients receiving continuous therapy, median PFS was 5.42 months (95% CI 2.76 – not estimable).

Of the patients eligible for intermittent therapy, the median age was 66 (range, 57–72; IQR 11). All five were male with ccRCC histology. All underwent prior nephrectomy and had IMDC intermediate-risk disease. Four of these patients had received only one prior therapy (3 sunitinib and 1 pazopanib) and one patient had received prior sunitinib followed by dalantercept/axitinib.

**Fig. 1:**
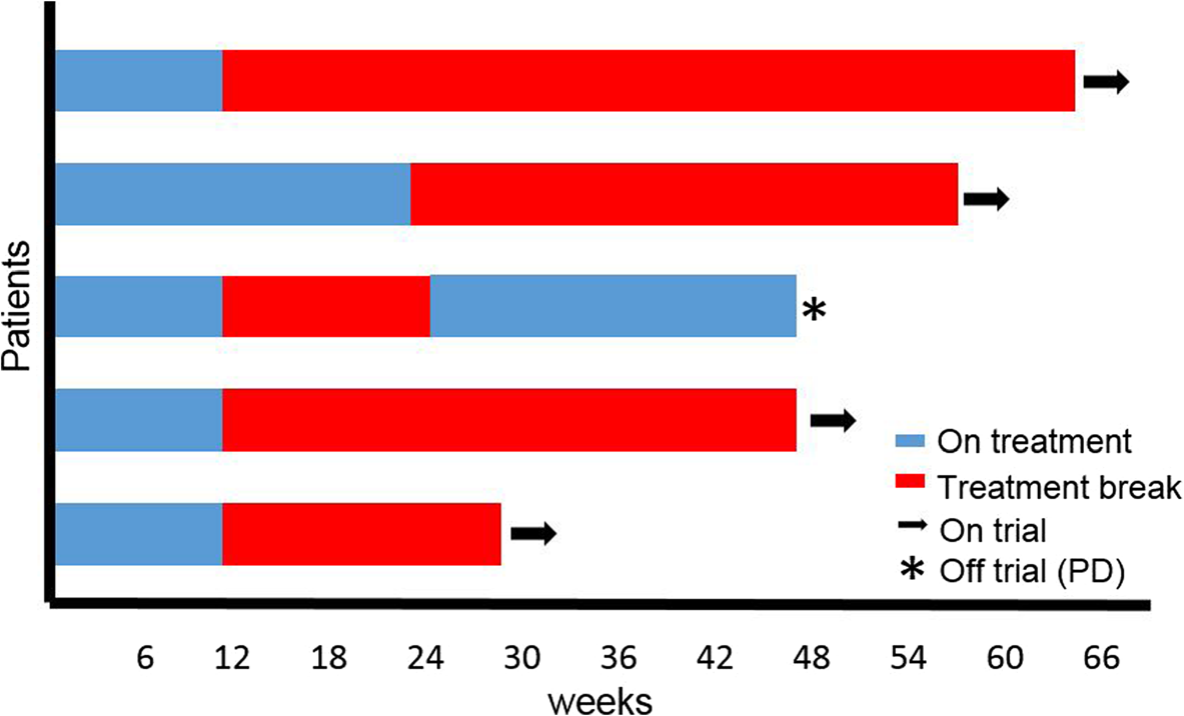
Duration on therapy and treatment-free intervals for patients in intermittent therapy phase (*n* = 5)

With a median follow-up of 48 weeks (IQR 8) for the patients who entered the intermittent therapy phase, only one patient restarted therapy (Fig. 1). At the first restaging scans, this patient initially had a 91% TB decrease and as such nivolumab was held per protocol. Therapy was restarted after a 12 weeks break given concerning tumor growth in non-target lesions. This patient eventually discontinued therapy 6 months following re-initiation of nivolumab due to the development of a new metastatic lesion in his liver. The four remaining patients have a sustained clinic response for a median of 34 weeks (range, 16–53; IQR 12) off therapy with a median sustained TB decrease of 46.5% (38–80%; IQR 16). No patients had RECIST-defined PD while on treatment break.

## Discussion

In this single-arm phase II trial, an intermittent nivolumab approach was implemented in which patients who achieved a pre-specified tumor burden decrease were taken off therapy and monitored by CT imaging. The key findings were that one-third of patients were eligible to stop therapy and all agreed. In addition, 4 of 5 patients who stopped therapy remain off therapy with a durable clinical response. The ORR in this trial (29%) is similar to that of the phase III CheckMate 025 trial (25%) that established nivolumab as a treatment option for patients with mRCC who had received prior anti-angiogenic therapy [1]. Patients in this study exhibited a longer median PFS (7.97 months) than the CheckMate 025 (4.6 months), likely a result of the small sample size and the five patients who entered the intermittent therapy phase.

The role of intermittent therapy in metastatic cancers has been well established in other malignancies which demonstrate that such stop-and-go approaches can improve toxicity and tolerability without limiting clinical outcomes [6, 7]. Extended breaks can result in decreased cumulative toxicity. Intermittent therapy can reduce cost of treatment as well as minimize patient and family time away from work thus reducing financial toxicity. This is of particular value as rising costs of healthcare and financial toxicity have been associated with poor adherence to medications and worse outcomes in cancer patients [8, 9].

In mRCC, multiple studies support the use of intermittent therapy in patients treated with VEGF-directed therapy [10–12]. Most recently, a prospective trial of intermittent sunitinib in which patients were granted extended treatment breaks following pre-specified tumor burden reduction also demonstrated feasibility of such an approach in mRCC. In that trial, 37 patients were enrolled with 20 patients entering the intermittent therapy phase. Of the 20 patients in the intermittent phase, the median PFS was 37.6 months with seven patients having prolonged treatment breaks ranging from 3.2 to 43.6 months. No patients had PD while on a treatment break [12].

Intermittent therapy approaches with an emphasis on extended treatment-free intervals (TFI) are especially critical with CPI therapy given data suggesting durable responses to immunotherapy even after treatment discontinuation [1–5, 13]. This was recently highlighted in an analysis of the phase III CheckMate214 trial in which ipilimumab/nivolumab demonstrated an overall survival benefit vs sunitinib in patients with treatment-naïve intermediate and poor risk mRCC [2, 5]. The TFI in patients who discontinued treatment was significantly longer in patients treated with ipilimumab/nivolumab compared to those treated with sunitinib (*p* < 0.0001). At 18 months following therapy discontinuation, 19% of patients treated with ipilimumab/nivolumab remained off of therapy compared to only 4% of sunitinib patients [5].

The primary endpoint for this trial was feasibility which was defined as the proportion of patients eligible for intermittent therapy who agreed to this approach. Given that the feasibility of an intermittent approach with immunotherapy in mRCC appears feasible (100% in this cohort), the results of this trial support the enrollment of patients into a subsequent cohort that will investigate the efficacy of intermittent ipilimumab/nivolumab as its primary endpoint.

There are inherent limitations to this study. For reasons mentioned above, the study was closed prior to complete accrual thus resulting in a small sample size available for analysis. The results herein are also from a highly specialized academic center. Despite these and other limitations, this prospective trial provides important evidence that intermittent approaches to immunotherapy treatment in mRCC patients are feasible.

## Conclusions

This prospective experience of intermittent nivolumab dosing in mRCC supports further investigation of intermittent immunotherapy dosing strategies in RCC. Ongoing trials of intermittent treatment will prospectively identify patients who can benefit from extended breaks without compromising clinical outcomes.
